# Dynamic Epicardial Contribution to Cardiac Interstitial c-Kit and Sca1 Cellular Fractions

**DOI:** 10.3389/fcell.2022.864765

**Published:** 2022-05-30

**Authors:** C. Pogontke, J. A. Guadix, A. M. Sánchez-Tévar, R. Muñoz-Chápuli, A. Ruiz-Villalba, J. M. Pérez-Pomares

**Affiliations:** ^1^ Department of Animal Biology, Faculty of Sciences, University of Málaga, Málaga, Spain; ^2^ Instituto Malagueño de Biomedicina (IBIMA)-Plataforma BIONAND, Universidad de Málaga, Málaga, Spain

**Keywords:** cardiac interstitium, epicardium, blood-borne cells, Sca1+ cells, c-Kit+ (CD117+) cells

## Abstract

**Background:** The cardiac interstitial cellular fraction is composed of multiple cell types. Some of these cells are known to express some well-known stem cell markers such as c-Kit and Sca1, but they are no longer accepted to be true cardiac stem cells. Although their existence in the cardiac interstitium has not been disputed, their dynamic throughout development, specific embryonic origin, and potential heterogeneity remain unknown. In this study, we hypothesized that both c-Kit^POS^ and Sca1^POS^ cardiac interstitial cell (CIC) subpopulations are related to the Wilms’ tumor 1 (Wt1) epicardial lineage.

**Methods:** In this study, we have used genetic cell lineage tracing methods, immunohistochemistry, and FACS techniques to characterize cardiac c-Kit^POS^ and Sca1^POS^ cells.

**Results:** Our data show that approximately 50% of cardiac c-Kit^POS^ cells are derived from the Wt1-lineage at E15.5. This subpopulation decreased along with embryonic development, disappearing from P7 onwards. We found that a large proportion of cardiac c-Kit^POS^ cells express specific markers strongly suggesting they are blood-borne cells. On the contrary, the percentage of Sca1^POS^ cells within the Wt1-lineage increases postnatally. In accordance with these findings, 90% of adult epicardial-derived endothelial cells and 60% of mEFSK4^POS^ cardiac fibroblasts expressed Sca1.

**Conclusion:** Our study revealed a minor contribution of the Wt1-epicardial lineage to c-Kit^POS^ CIC from embryonic stages to adulthood. Remarkably, a major part of the adult epicardial-derived cell fraction is enriched in Sca1, suggesting that this subpopulation of CICs is heterogeneous from their embryonic origin. The study of this heterogeneity can be instrumental to the development of diagnostic and prognostic tests for the evaluation of cardiac homeostasis and cardiac interstitium response to pathologic stimuli.

## Introduction

The vertebrate heart is composed of a plethora of different cell types. Around 70% of these cells reside in the cardiac interstitium (CI), the extracellular space between cardiomyocytes (Bergmann et al., 2015; Pinto et al., 2016). All these cells are jointly referred to as cardiac interstitial cells (CICs) and are known to play an essential role in myocardial embryonic development, and adult homeostasis (Krenning et al., 2010; Pérez-Pomares and de La Pompa, 2011; Pogontke et al., 2019), and in the adaptive responses of the heart to pathological conditions (Takeda et al., 2011; Ruiz-Villalba et al., 2015).

The CIC population is highly heterogeneous. It comprises multiple cell types such as endothelial cells (ECs), vascular smooth muscle cells (vSMCs), cardiac fibroblasts (CFs), pericytes, and circulating cells among others (Ruiz-Villalba et al., 2015; Pinto et al., 2016; Sampaio-Pinto et al., 2020). These different cellular pools are not, however, equally represented. Indeed, ECs, which approximately represent 58% of all CICs, are very abundant. This finding is in accordance with extensive myocardial vascularization by coronary vessels, including arteries, veins, and a massive capillary bed (Tomanek et al., 2010). Furthermore, the coronary endothelium has a key scaffolding role during embryonic interstitium formation (Pogontke et al., 2019) since coronary EC interaction with vSMCs, pericytes, and CFs is necessary for the building of periendothelial coronary domains. Moreover, due to the cellular complexity, a niche role for the periendothelial coronary milieu has been proposed (Fioret et al., 2014; Pogontke et al., 2019). Unlike EC, other cell types are far less frequent in the CI. For example, the small numbers of blood-borne cells have been described to permanently or transiently reside in the adult cardiac interstitium in the absence of evident pathological stimuli (Epelman et al., 2014; Molawi et al., 2014). The functions played by these cells in the healthy heart are poorly known, and data on the specific diversity and the spatiotemporal distribution of these cells are scarce (Pinto et al., 2016; Farbehi et al., 2019).

Other sparse cells holding an intrinsic multipotent differentiation potential were reported to be present in the cardiac interstitium. These cells, frequently dubbed cardiac stem cells (CSCs), were thought to be the origin of *de novo* cardiomyocyte differentiation in the adult heart. A bone marrow origin for these cells was discarded by some authors based on the absence of CD45 expression (Limana et al., 2005; Bearzi et al., 2007) but not by other researchers (Zhou et al., 2010). CSCs were originally identified based on their characteristic expression of molecules like Bmi1, Abcg2, Isl-1, Sca1, or c-Kit (Martin-Puig et al., 2012; Anversa et al., 2013), all of which are known to be expressed by other organ-resident, well-characterized stem or progenitor cells (Bradfute et al., 2005; Thoren et al., 2008). Of these, c-Kit- and Sca1-expressing CSCs have been the most widely studied. Both molecules are associated with stem/progenitor cell properties and are often considered markers for these cell types. The membrane-bound stem cell factor (SCF) receptor c-Kit (a.k.a. CD117) is expressed by hematopoietic cells but also by other cell types, many of which do not display stem/progenitor cell properties (Lennartsson and Rönnstrand, 2012; Liang et al., 2013). Similarly, Sca1 (Stem Cell Antigen 1) is expressed in different cell populations, including blood and cancer cells, and only a fraction of Sca1-expressing cells has been unambiguously shown to represent a stem/progenitor cell fraction (Holmes and Stanford, 2007).

As indicated, since 2003, cardiac c-kit^POS^ cells have been thought to be a self-renewing, clonogenic, and multipotent population of CSC. These cells were described to differentiate into a minimum of three different cardiogenic cell lineages (myocytes, smooth muscle cells, and endothelial cells) *in vitro* (Beltrami et al., 2003) and, when grown in non-adherent *in vitro* assays, c-Kit^POS^ CSCs were shown to form cardiospheres (Vicinanza et al., 2017). Most importantly, these cells were characterized, isolated, expanded, and used in cell-based experimental therapies to treat the diseased heart (Gude and Sussman, 2018; Keith and Bolli, 2015). Intensive recent research, however, has revealed that the myocardiogenic potential of these c-kit^POS^ CSCs is negligible (Van Berlo et al., 2014; Sultana et al., 2015), so their actual multipotency has been refuted. As for cardiac Sca1^POS^ cells, different studies have shown that they robustly differentiate into endothelial cells *in vitro* after treatment with VEGF or PDGF BB (CD31^POS^, vWF^POS^, caveolin^POS^), and form endothelial tubules when cultured in 3D matrices (e.g., Matrigel) (Takamiya et al., 2011; Wang et al., 2006). More recently, these cells have also been associated with cardiac endothelial cells and fibroblasts *in vivo* using a transgenic model for lineage tracing (Tang et al., 2018).

Regardless of their multipotent potential, the existence of cardiac cells expressing molecular markers classically associated with stem cells such as c-Kit and Sca1 has not been disputed. However, their dynamics along with embryonic development and adulthood, specific origin, nature, and function remain obscure. In this work, we aimed to progress in the characterization of these elusive cells by the evaluation of a possible epicardial origin for, at least, part of them, also considering the bone marrow/circulation as a plausible source for cardiac c-Kit^POS^ and Sca1^POS^ cells. This working hypothesis is supported by the well-known contribution of the embryonic epicardium to the adult heart resident CICs pool (Ruiz-Villalba et al., 2015; Volz et al., 2015; Cano et al., 2016). In order to tackle this objective, permanent genetic tracing of epicardial cell derivatives has been carried out taking advantage of strong expression of the *Wilms’ tumor suppressor gene* (*Wt1*) in embryonic epicardial cells and by carefully analyzing tissues using both immunohistochemistry and FACS.

## Materials and Methods

### Animal Models

All animals used in this study were handled in compliance with institutional and European Union guidelines for animal care and welfare under a specific experimental procedure approved by the Committee on Ethics of Animal Experiments of the University of Málaga/BIONAND.

For lineage-tracing studies, homozygous Tg(WT1-cre)^#Jbeb^ line (Wt1Cre from now on), in which an IRES/GFP-Cre cassette was inserted 17 bp downstream of the translation stop site of the Wilms' Tumor Gene 1 (Wt1) gene was used as driver line (Del Monte et al., 2011). The B6.129X1-Gt(ROSA)26Sor^tm1(EYFO)Cas/J^ line (Rosa26R-eYFP from now on), that contains the enhanced yellow fluorescent protein gene downstream of a loxP-flanked stop sequence, was used as reporter line (Srinivas et al., 2001). Both lines were crossed in homozygosis, obtaining Wt1Cre-YFP^POS^ mice in which Wt1-driven Cre activity mediates the excision of the LoxP-flanked STOP sequence in R26R mice, activating permanent reporter enhanced yellow fluorescent protein (eYFP) expression in the *Wt1* ^POS^ cell linage.

For conditional tracing studies, homozygous Wt1^tm2(cre/ERT2)Wtp/J^ mice (Wt1Cre/ERT2 from now on), in which exon 1 of the Wt1 locus was replaced by a CreERT2 fusion gene, were crossed with homozygote Rosa26R-eYFP, obtaining Wt1Cre/ERT2-YFP^POS^, where eYFP expression is permanently activated in the Wt1^POS^ cell lineage from the moment of the chemical induction using tamoxifen (Sigma) (Zhou et al., 2008). Tamoxifen was dissolved in corn oil (Sigma) at 1 mg/ml and 50 µl were injected into newborns by intragastric injection at P1, P2, and P3 (Lizen et al., 2015). An equivalent volume of corn oil was administered to control animals. The processing of the pup’s hearts was performed at P7. The embryos were staged considering the moment of vaginal plug observation, which was designated as E0.5.

### Flow Cytometry

Wt1Cre-YFP^POS^ and Wt1Cre/ERT2-YFP^POS^ hearts were dissociated through two sequential steps (10 min) in 37°C pre-warmed liberase TH (0.125 mg/ml, Roche). After complete digestion, the samples were filtered to eliminate cardiomyocytes (Cell Strainer 40 μm filters, BD) and red blood cells were lysed (Roche). The cell samples were then incubated in the proper primary antibodies (see Supplementary Table S1) diluted in 2% BSA, 1 mM EDTA in PBS for 15 min, using non-immune IgGs or PBS as negative controls (Supplementary Figure S1). For the exclusion of non-viable cells in flow cytometry analysis, 7AAD (EBioscience) was employed. The results were recorded in a Gallios flow cytometer (Beckman Coulter) and analyzed using the Kaluza software.

### Immunohistochemistry

Embryonic (E13.5, E15.5, and E18.5), neonate (P1, P7), and adult hearts (8–12 week-old) were excised and washed in PBS, fixed in 4% fresh paraformaldehyde, cryoprotected in sucrose and frozen in liquid N2-cooled isobutanol. Ten-micrometer cryosections were rehydrated in PBS, non-specific IgG binding sites blocked with 10% horse serum, 1.5% BSA, and 0.1% Triton X-100 in TPBS and incubated overnight (ON) in the proper primary antibodies (Supplementary Table S1). Negative controls were performed incubating with fluorochrome-conjugated secondary antibodies without primary antibodies (Supplementary Table S2). The cell nuclei were counterstained with 4′,6-diamidino-2-phenylindole (DAPI) (Sigma). All images were captured in a Leica SP5 laser confocal microscopy.

### Statistical Analysis

For FACS studies, the statistical significance was analyzed by the Student’s t-test, and shown as mean plus standard deviation (*p* < 0.05). Three biological replicates were used in embryonic samples (5 littermates were pooled for each replicate for E15.5, and 3 littermates for E18.5). Three individual samples were used for each neonatal time point and eight for adults, without significant differences derived from the gender.

## Results

### c-Kit^POS^ Cardiac Interstitial Cells Do Not Belong to the Wt1 Lineage

**FIGURE 1:**
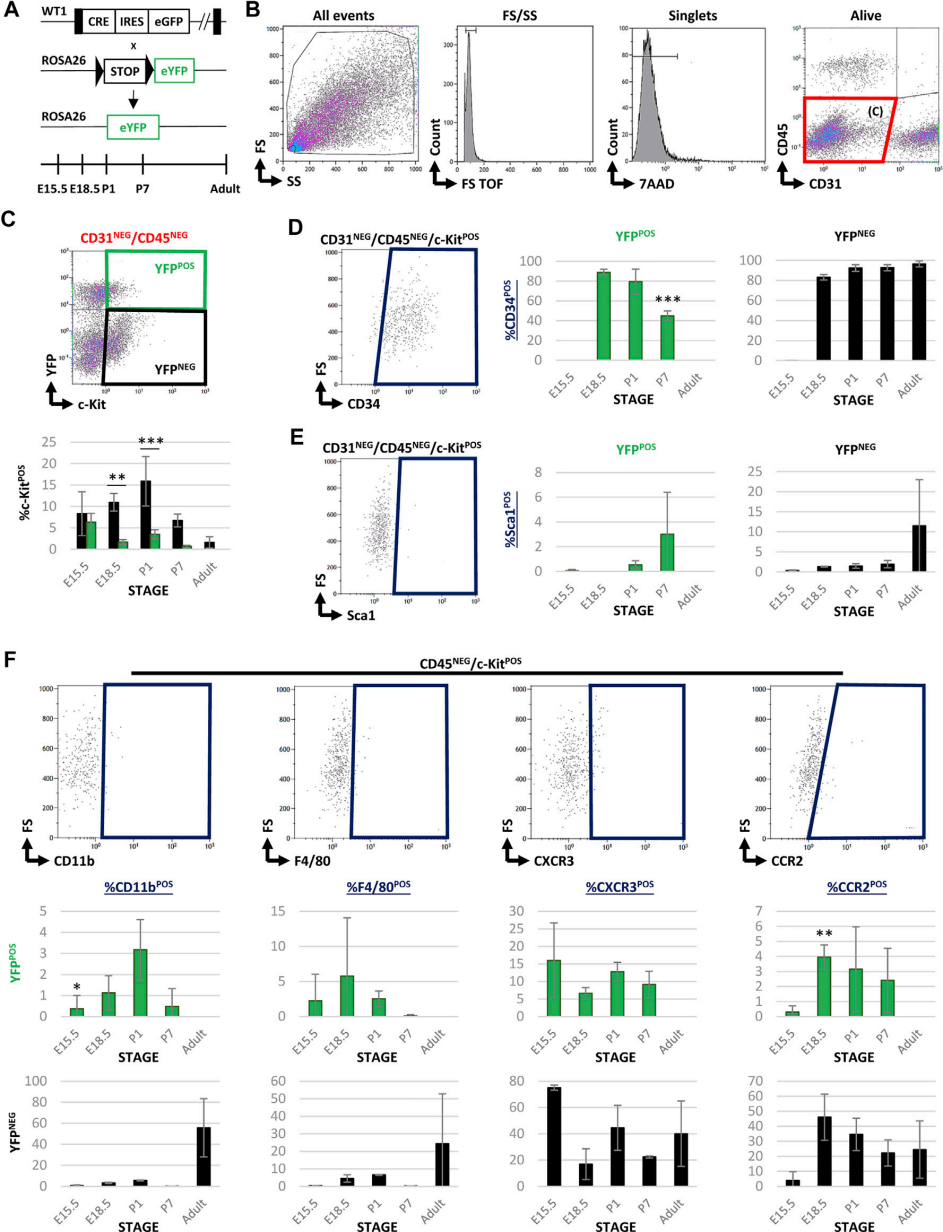
FACS characterization of cardiac c-Kit^POS^ cells during development and adulthood in Wt1Cre-YFP mice. **(A)** Wt1Cre-YFP mice were generated as shown. The tissue samples were obtained in different time points during embryonic development (E15.5 and E18.5), postnatal stages (P1 and P7), and adult hearts. **(B)** Representative dot blots and cytograms obtained were obtained from the FACS analysis showing the sequential gating used for the characterization of CD31^NEG^/CD45^NEG^ cardiac interstitial cells. **(C)** Representative dot blot was used for the characterization of c-Kit and YFP expression within the subpopulation of CD31^NEG^/CD45^NEG^ cells. The same gating was applied for all the different stages included in this study (above). The bar plot representing the percentages of CD31^NEG^/CD45^NEG^/c-Kit^POS^ cells present in all stages considered; YFP^NEG^ (black bars), YFPPOS (green bars). **(D)** Representative dot blot for the characterization of CD34^POS^ cells within the CD31^NEG^/CD45^NEG^/c-Kit^POS^ subpopulation (left). The bar plots indicated the percentage of CD34^POS^ cells found in the CD31^NEG^/CD45^NEG^/c-Kit^POS^/YFP^POS^ (green bars, middle) and YFP^NEG^ (black bars, right) subpopulations, respectively. **(E)** Representative dot blot for the characterization of Sca1^POS^ cells within the CD31^NEG^/CD45^NEG^/c-Kit^POS^ subpopulation (left). The bar plots showed the percentage of Sca1^POS^ cells in the CD31^NEG^/CD45^NEG^/c-Kit^POS^/YFP^POS^ (green bars, middle) and YFP^NEG^ (black bars, right) subpopulations, respectively. **(F)** Representative dot blots showing the characterization of several markers for circulating cells (CD11b, CCR2, F4/80, and CXCR3) within the CD45^NEG^/c-Kit^POS^ cardiac interstitial cell subpopulation. The bar plots illustrated the percentage of cells stained with these surface markers in the CD45^NEG^/c-Kit^POS^/YFP^POS^ (green bars) and YFP^NEG^ (black bars) subpopulations, respectively. Abbreviations: CCR2, C-C motif chemokine receptor 2; CD11b, cluster of differentiation 11; CD31/PECAM1 = platelet and endothelial cell adhesion molecule 1; CD34, cluster of differentiation 34; CD45, protein tyrosine phosphatase receptor type C; c-Kit, KIT proto-oncogene receptor tyrosine kinase; CXCR3, C-X-C motif chemokine receptor 3; F4/80, EGF-like module-containing mucin-like hormone receptor-like 1; FS, forward scatter; Sca1, stem cell antigen-1; SS, side scatter; YFP, yellow fluorescent protein. Each experimental group includes the following biological replicates: 3 litters for embryonic stages (5 or 3 embryos peer biological replicate for E15.5 and E18.5, respectively); 3 individual, biological replicates for P1 and P7; 8 animals, biological replicates for adults. *p-value ≤0.05; **p-value ≤0.01; ***p-value ≤0.005.

Wt1Cre-YFP transgenic mice, resulting from the crossing of the Wt1Cre line with the reporter Rosa26R-eYFP one, allow for the permanent tracing of epicardial and putative epicardial-derived cells from development to the adulthood (Srinivas et al., 2001) (Figure 1A). In order to characterize Wt1 lineage-derived cardiac interstitial cells (CICs) population, we first FACS screened the colocalization of c-Kit and YFP in the CD31^NEG^/CD45^NEG^ interstitial cell fraction. Our analysis showed that the CD31^NEG^/CD45^NEG^ CIC pool, which excludes endothelial and circulating cells, contains a low c-Kit^POS^ CICs number (between 1.6 and 22%, see Table 1). Notwithstanding, the proportion of CD31^NEG^/CD45^NEG^/c-Kit^POS^ significantly varies through time, peaking at P1 (around 22% of the total CD31^NEG^/CD45^NEG^ CICs) (Table 1). At E15.5 almost 14% of CD31^NEG^/CD45^NEG^ CICs co-expressed c-Kit. Of them, almost half were YFP^POS^ (43%, see Figure 1C; Table 1), suggesting a significant contribution of Wt1-lineage to the CD31^NEG^/CD45^NEG^ CIC population (6.33 ± 2.06% of CD31^NEG^/CD45^NEG^ were c-Kit^POS^/YFP^POS^ and 8.31 ± 5.13% c-Kit^POS^/YFP^NEG^) (Table 1). From E15.5 until P1, the proportion of CD31^NEG^/CD45^NEG^/c-Kit^POS^/YFP^POS^ cells was reduced to 10% of the total c-Kit^POS^ CICs present in the heart. Finally, no CD31^NEG^/CD45^NEG^/c-Kit^POS^/YFP^POS^ CICs were detected from P7 onwards (Figure 1C; Table 1).

**TABLE 1:** Percentage of c-Kit^POS^ cells included in both YFP^POS^ and YFP^NEG^ fractions into the different characterized subpopulations (mean ± s.e.m.).

CD31^NEG^/CD45^NEG^				
	YFP	c-Kit^POS^	c-Kit^POS^/CD34^POS^	c-Kit^POS^/Sca1^POS^
E15.5	+	6.33 ± 2.06	0 ± 0	0.06 ± 0.1
	−	8.31 ± 5.13	0.09 ±0.12	0.34 ± 0.17
E18.5	+	1.70 ± 0.56	88.75 ± 3.29	0 ± 0
	−	10.97 ± 2.05	83.14 ± 2.69	1.30 ± 0.12
P1	+	3.65 ± 1.32	79.42 ± 12.73	0.54 ± 0.33
	−	18.78 ± 5.79	92.46 ± 3.35	1.47 ± 0.54
P7	+	0.67 ± 0.29	44.81 ± 5.01	3.02 ± 3.38
	−	6.72 ± 1.49	92.76 ± 3.04	1.93 ± 0.92
Adult	+	0 ± 0	Na	Na
	−	1.61 ± 1.31	96.49 ± 2.97	11.48 ± 11.51

CD45^NEG^/c-Kit^POS^					
	YFP	CD11b^POS^	F4/80^POS^	CXCR3^POS^	CCR2^POS^
E15.5	+	0.37 ± 0.64	2.24 ± 3.80	16.01 ± 10.72	0.30 ± 0.41
	−	0.6 ± 0.84	0.28 ± 0.08	75.06 ± 2.01	3.93 ± 5.81
E18.5	+	1.12 ± 0.82	5.78 ± 8.32	6.63 ± 1.66	3.96 ± 0.82
	−	3.30 ± 0.53	4.46 ± 2.09	16.81 ± 11.73	46.09 ± 15.35
P1	+	3.18 ± 1.43	2.55 ± 1.10	12.83 ± 2.65	3.15 ± 2.82
	−	5.60 ± 0.45	6.53 ± 0.16	44.57 ± 17.16	34.56 ± 10.78
P7	+	0.49 ± 0.84	0.11 ± 0.18	9.18 ± 3.76	2.41 ± 2.13
	−	0.19 ± 0.04	0.19 ± 0.04	22.40 ± 0.77	22.26 ± 8.68
Adult	+	Na	Na	na	na
	−	55.69 ± 27.77	24.33± 28.49	40.05 ± 24.89	40.05 ± 24.89

	YFP	CD45^POS^/c-Kit^POS^	CD31^POS^/c-Kit^POS^
E15.5	+	0 ± 0	0.64 ± 0.52
	−	65.23 ± 12.87	10.94 ± 5.75
E18.5	+	0.12 ± 0.21	0.12 ± 0.05
	−	25.20 ± 1.87	5.05 ± 0.53
P1	+	0.76 ± 0.56	0.14 ± 0.08
	−	33.21 ± 4.11	2.26 ± 0.98
P7	+	0.56 ± 0.78	0 ± 0
	−	16.76 ± 3.53	0.01 ± 0.02
Adult	+	0.35 ± 0.58	0.07 ± 0.13
	−	12.90 ± 7.70	1.54 ± 2.17

In order to characterize both subpopulations of c-Kit^POS^ CICs (YFP^POS^ *vs*. YFP^NEG^), we analyzed the presence of CIC surface markers often associated with multipotency in literature, such as CD34 and Sca1. Both the YFP^POS^ and YFP^NEG^ cell subpopulations showed a similar proportion of CD34 expressing cells along with embryonic development (Figure 1D). The percentage of CD31^NEG^/CD45^NEG^/c-Kit^POS^/YFP^POS^/CD34^POS^ CICs is however reduced at P7, disappearing in the adult (Figure 1D). The percentage of Sca1^POS^ cells in the c-Kit^POS^/YFP^POS^ fraction was negligible, and in the c-Kit^POS^/YFP^NEG^ one was highly variable (11.48 ± 11.51%, Figure 1E), indicating that both subpopulations are different cell types.

After the preliminary evaluation of c-Kit^POS^ CICs relationship with the Wt1 cell lineage, we decided to explore a potential alternative origin for these cells. Based on the published evidence of blood-borne cell contribution to the cardiac interstitium (Zhang et al., 2006; Pinto et al., 2016; Sampaio-Pinto et al., 2020), we investigated whether CD45, a pan-leukocyte marker for circulating cells, was expressed (Supplementary Figure S2A). Our analysis showed that the number of CD45^POS^/c-Kit^POS^ cells decreased from 65% to 13% between E15.5 and adulthood (Table 1). Surprisingly, a significant number of CD45^NEG^/c-Kit^POS^/YFP^NEG^ CICs were enriched in cell surface markers classically associated with different subpopulations of circulating cells, such as myeloid cells (CD11b), monocyte/macrophages lineage (CCR2), macrophages (F4/80), and some lymphoid cell types (CXCR3), strongly suggesting an extracardiac origin for these cells (Figure 1F). Some of these circulating cell markers were also present in CD45^NEG^/c-Kit^POS^/YFP^POS^ CICs in perinatal stages, in accordance with the described expression of Wt1 in a restricted population of adult blood cells (Alberta et al., 2003; King-Underwood et al., 2005). Finally, we found a reduced contribution of c-Kit^POS^ cells into CD31^POS^ endothelium from E15.5 (around 11%) to adulthood (around 1.6%) (Supplementary Figure S2B). In both the CD45^POS^ and the CD31^POS^/c-Kit^POS^ subpopulations, the co-expression with YFP was restricted.

**FIGURE 2:**
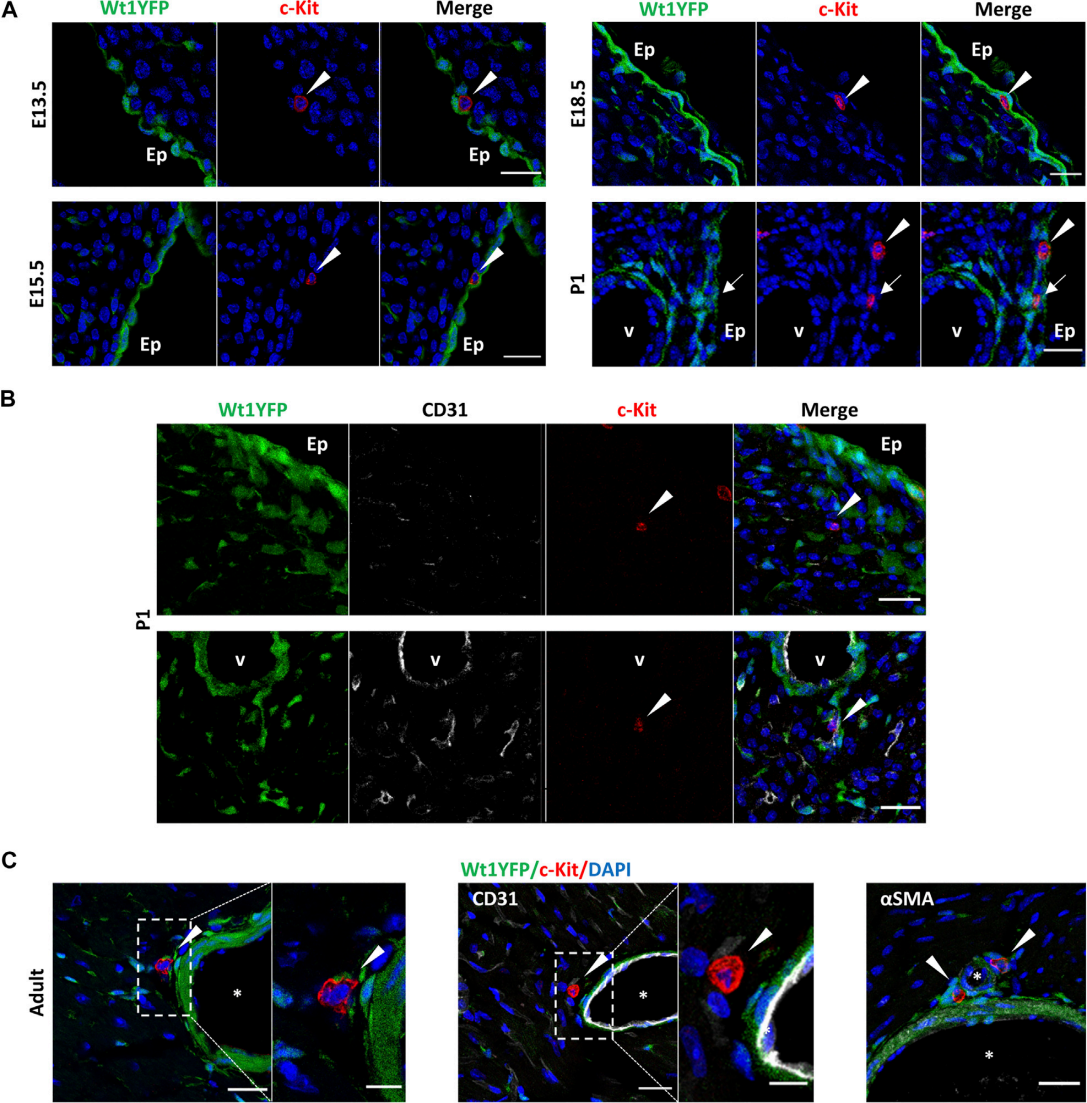
Dynamic of the topological distribution of c-Kit^POS^ cells in the embryonic, post-natal, and adult heart. Immunolocalization of c-Kit in the representative sections of Wt1Cre-YFP hearts, in which cells derived from the Wt1 epicardial lineage are constitutively expressed YFP (green). **(A)** c-Kit^POS^/YFP^NEG^ cells (red) are present in the (sub)epicardium from E13.5 to postnatal stages (arrowheads). At P1, a few c-Kit^POS^/YFP^POS^ cells (yellow) were detected in this region. **(B)** c-Kit^POS^/YFP^NEG^ cells (red, arrowheads) were found in the cardiac interstitium and close to the coronary vessels (P1). **(C)** In the adult, c-Kit^POS^/YFP^NEG^ cells (red, arrowheads) were localized next to the adventitia of large coronary vessels, close to αSMA^POS^/YFP^POS^ smooth muscle cells (light green). Abbreviations: αSMA, alpha-smooth muscle actin; CD31/PECAM1, platelet, and endothelial cell adhesion molecule 1; c-Kit, KIT proto-oncogene receptor tyrosine kinase; DAPI, 4′,6-diamidino-2-fenilindol; Ep, epicardium; YFP, yellow fluorescent protein. V, the lumen of coronary vessels. Scale bars: 25 μm **(A,B)**; magnifications in C: 10 μm.

To validate these FACS data as well as to identify the specific anatomical distribution of c-Kit^POS^ cells, we analyzed the localization of c-Kit in the heart using immunohistochemistry. We could not find any c-Kit^POS^ cells in any embryonic hearts before 13.5 days of development (data not shown). At E13.5, most c-Kit^POS^ cell co-expressed CD45 (arrows in Supplementary Figure S2C), and a few of them CD31 (arrows in Supplementary Figure S1D) by immunohistochemistry. From E13.5 onwards both CD45^POS^/c-Kit^POS^ and CD45^NEG^/c-Kit^POS^ CICs were always located in the subepicardial of embryonic ventricles (Figure 2A). No c-Kit^POS^/YFP^POS^ cells were found in any of the analyzed hearts (*n* = 2 per stage), except at P1 (arrows in Figure 2). It is important to note that most postnatal c-Kit^POS^ cells were located either close to coronary vessels or incorporated into the surface of the coronary adventitial layer (Figure 2B).

Taken together, our results clearly demonstrated that the presence of c-Kit^POS^ cells in the cardiac interstitium is highly dynamic through development and adulthood. Moreover, c-Kit^POS^ CICs are a heterogeneous population of cells from their developmental origin point of view, with a minimal contribution coming from Wt1 lineage.

### Wt1 Cardiac Lineage Significantly Contributes to the Interstitial Sca1^POS^ Cell Fraction

**FIGURE 3:**
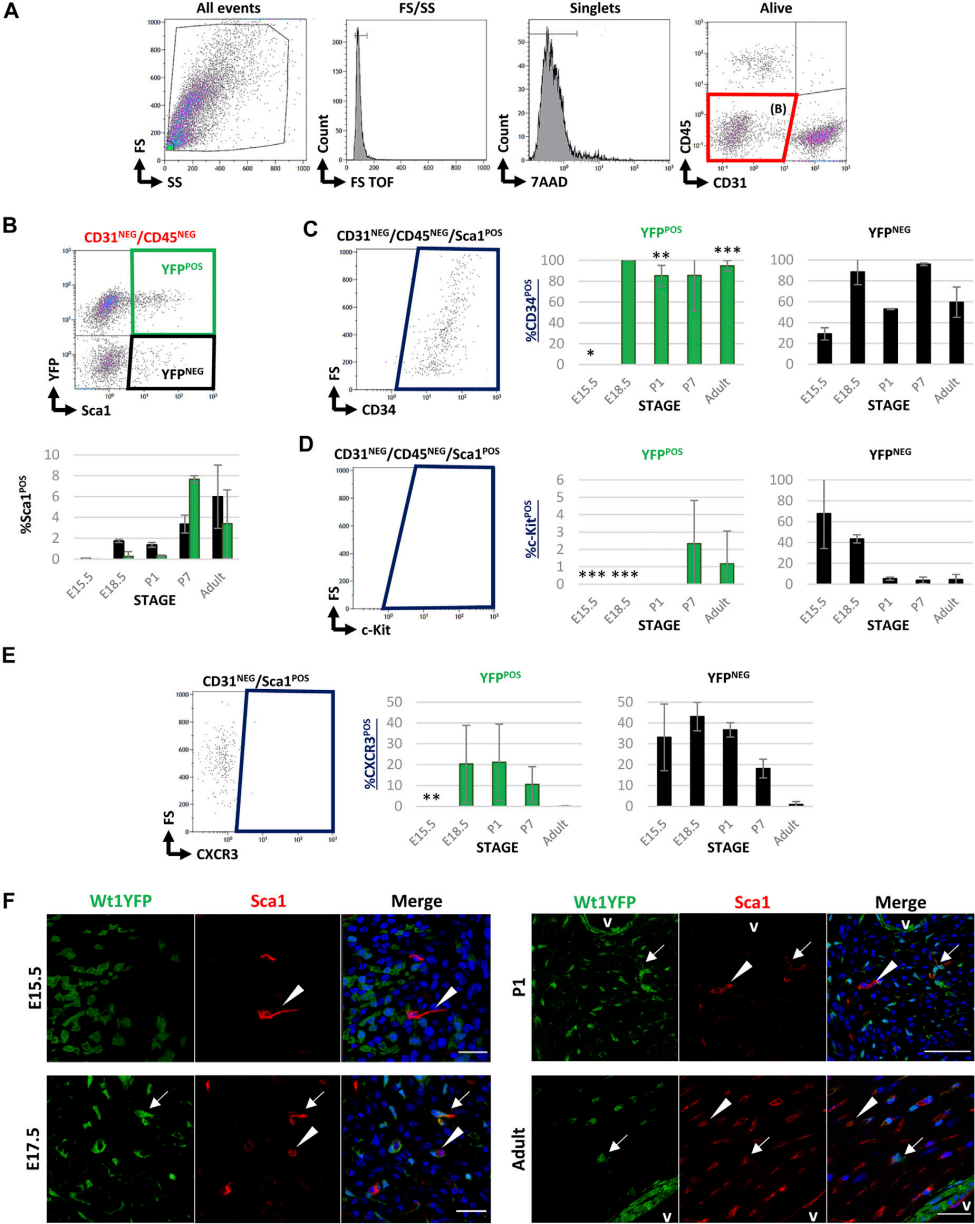
FACS characterization of cardiac Sca1^POS^ cells during development and adulthood in Wt1Cre-YFP mice. **(A)** Representative dot blots and cytograms from the FACS analyses show the sequential gating used for the characterization of CD31^NEG^/CD45^NEG^ cardiac interstitial cells. **(B)** Representative dot blot of the gating used for the characterization of Sca1 and YFP within the CD31^NEG^/CD45^NEG^ cells subpopulation. This same gating was used in all the stages considered in this study (upper row). The bar plots representing the percentages of CD31^NEG^/CD45^NEG^/Sca1^POS^ cells present in all the stages considered in this work (E15.5, E18.5, P1, P7, and adult). YFP^POS^ (green bars) and YFP^NEG^ (black bars) are identified (lower row). **(C)** Representative dot blot for the characterization of cardiac CD34^POS^ cells within the CD31^NEG^/CD45^NEG^/Sca1^POS^ subpopulation (left). The bar plots illustrated the percentage of CD34^POS^ cells within the CD31^NEG^/CD45^NEG^/Sca1^POS^/YFP^POS^ (green bars, middle) or YFP^NEG^ (black bars, right) subpopulations, respectively. **(D)** Representative dot blot for the characterization of c-Kit^POS^ cells within the CD31^NEG^/CD45^NEG^/Sca1^POS^ subpopulation (left). The bar plots show the percentage of c-Kit^POS^ cells within the CD31^NEG^/CD45^NEG^/Sca1^POS^/YFP^POS^ (green bars, middle) or YFP^NEG^ (black bars, right) subpopulations, respectively. **(E)** Representative dot blot for the characterization of c-KitPOS cells within the CD31NEG/Sca1POS subpopulation (left). The bar plots show the percentage of c-KitPOS cells within the CD31^NEG^/Sca1^POS^/YFP^POS^ (green bars, middle) or YFPNEG (black bars, right) subpopulations, respectively. **(F)** Immunolocalization of SCA1 staining in the representative sections from E13.5, 17.5, P1, and adult Wt1Cre-YFP hearts. SCA1^POS^/YFP^NEG^ (red, arrowheads) and SCA1^POS^/YFP^POS^ cells (yellow, arrows) home at the cardiac interstitium. Abbreviations: CD31/PECAM1, platelet, and endothelial cell adhesion molecule 1; CD34, cluster of differentiation 34; CD45, protein tyrosine phosphatase receptor type C; c-Kit, KIT proto-oncogene receptor tyrosine kinase; DAPI, 4′,6-diamidino-2-fenilindol; FS, forward scatter; Sca1, stem cell antigen-1; SS, side scatter; V, lumen of coronary vessels; YFP, yellow fluorescent protein. Each experimental group includes the following biological replicates: 3 litters for embryonic stages (5 or 3 embryos peer biological replicate for E15.5 and E18.5, respectively); 3 individual, biological replicates for P1 and P7; 8 animals, biological replicates for adults). Scale bars: 25 μm *p value ≤0.05; **p value ≤0.01; ***p value ≤0.005.

To evaluate the contribution of Wt1-derived cells to the cardiac Sca1^POS^ cell population, we performed FACS at the same stages selected for the study of c-Kit^POS^ CICs (Figure 1A) using the same gating strategy (Figure 3A). Sca1 was detected in a small percentage of CD31^NEG^/CD45^NEG^ CICs only, although the amount of Sca1^POS^ cells significantly varied over time (Table 2). During developmental and perinatal stages, Sca1^POS^ CICs represent between the 0%–2% of CD31^NEG^/CD45^NEG^ CICs but importantly increased between P1 and P7. The majority of Sca1^POS^ CICs derived from the epicardial lineage at P7 (7.65 ± 0.35% of CD31^NEG^/CD45^NEG^ were Sca1^POS^/YFP^POS^ and 3.37 ± 0.86% were Sca1^POS^/YFP^NEG^), although this contribution was reduced in the adult (Figure 3B; Table 2). In order to characterize the Sca1^POS^ CICs subpopulation in detail, we first checked for the expression of CD34. The percentage of CD31^NEG^/CD45^NEG^/Sca1^POS^/YFP^POS^ CICs expressing CD34 remained relatively stable from E18.5 onward. In contrast, the percentage of CD31^NEG^/CD45^NEG^/Sca1^POS^/YFP^NEG^/CD34^POS^ CICs was highly dynamic along the different stages (Figure 3C). Regarding the coexpression of c-Kit and Sca1, CD31^NEG^/CD45^NEG^/Sca1^POS^/c-Kit^POS^/YFP^POS^ CICs were detected at P7 only (Figure 3D). CD31^NEG^/CD45^NEG^/Sca1^POS^/c-Kit^POS^/YFP^NEG^ CICs were abundant at E15.5, but their number sharply decreased later on.

**TABLE 2:** Percentage of Sca1^POS^ cells included in both YFP^POS^ and YFP^NEG^ fractions into the different characterized subpopulations (mean ± s.e.m.).

CD31^NEG^/CD45^NEG^				
	YFP	Sca1^POS^	Sca1^POS^/CD34^POS^	Sca-1^POS^/c-kit^POS^
E15.5	+	0 ± 0.01	0 ± 0	0 ± 0
	−	0.06 ± 0.05	29.17 ± 5.89	67.78 ± 33.39
E18.5	+	0.27 ± 0.46	100 ± 0	0 ± 0
	−	1.74 ± 0.15	88.66 ± 12.19	43.43 ± 3.97
P1	+	0.33 ± 0.08	85.28 ± 9.87	0 ± 0
	−	1.38 ± 0.22	52.91 ± 0.55	5.36 ± 1.41
P7	+	7.65 ± 0.35	85.53 ± 34.14	2.33 ± 2.48
	−	3.37 ± 0.86	96.02 ± 1.18	3.83 ± 3.05
Adult	+	3.38 ± 3.27	94.78 ± 5.02	0 ± 0
	−	5.99 ± 3.03	59.51 ± 14.55	4.55 ± 4.78

	YFP	CD45^POS^/Sca1^POS^	CD31^POS^/Sca1^POS^
E15.5	+	0 ± 0	0.02 ± 0.02
	−	2.6 ± 1.68	0.84 ± 0.57
E18.5	+	0.12 ± 0.21	3.54 ± 0.6
	−	13.25 ± 1.34	30.48 ± 2.34
P1	+	0.58 ± 0.61	12.24 ± 1.20
	−	25.47 ± 3.33	60.84 ± 2.57
P7	+	0.89 ± 0.49	11.80 ± 4.82
	−	28.70 ± 3.19	81.61 ± 5.11
Adult	+	1.78 ± 2.01	4.22 ± 1.59
	−	11.36 ± 6.87	88.21 ± 2.68

Further evaluation of the samples revealed that the percentage of CD45^POS^/Sca1^POS^ CICs increased from E15.5 to P7 and then decreased in the adult (Supplementary Figure S3A; Table 2). Remarkably, more than 70% of Sca1^POS^ CICs coexpressed CD31 after birth (up to 90% in the adult); most of these cells were YFP^NEG^ (Supplementary Figure S3B; Table 2).

To validate the FACS analysis and map the cardiac location of Sca1^POS^ cells, we performed immunohistochemical analysis. Quite differently from our c-Kit immunohistochemical screening, Sca1^POS^ CICs were found to be distributed throughout all the cardiac ventricular interstitium. Furthermore, Sca1^POS^/YFP^POS^ CICs were identified in different stages (arrows in Figure 3E). In the adult, the majority of Sca1 colocalizes with *Griffonia simplicifolia* lectin (IB4), strongly suggesting an endothelial nature for these cells (arrowheads in Supplementary Figure S3C). Some of these Sca1^POS^ endothelial cells were also found to be YFP^POS^ (arrows in Supplementary Figure S3C).

In summary, our data demonstrated that Sca1^POS^ cells represent a low percentage of CICs with a heterogeneous cellular origin, including Wt1 lineage. Interestingly, these cells are mainly found in postnatal and adult stages, and are closely related to the cardiac endothelial lineage, suggesting a potential difference in this cell type in terms of cell proliferation, migration, and differentiation.

### Majority of Cardiac Wt1 Lineage-Derived Endothelial Cells and Fibroblasts Express Sca1 in the Adult Heart

**FIGURE 4:**
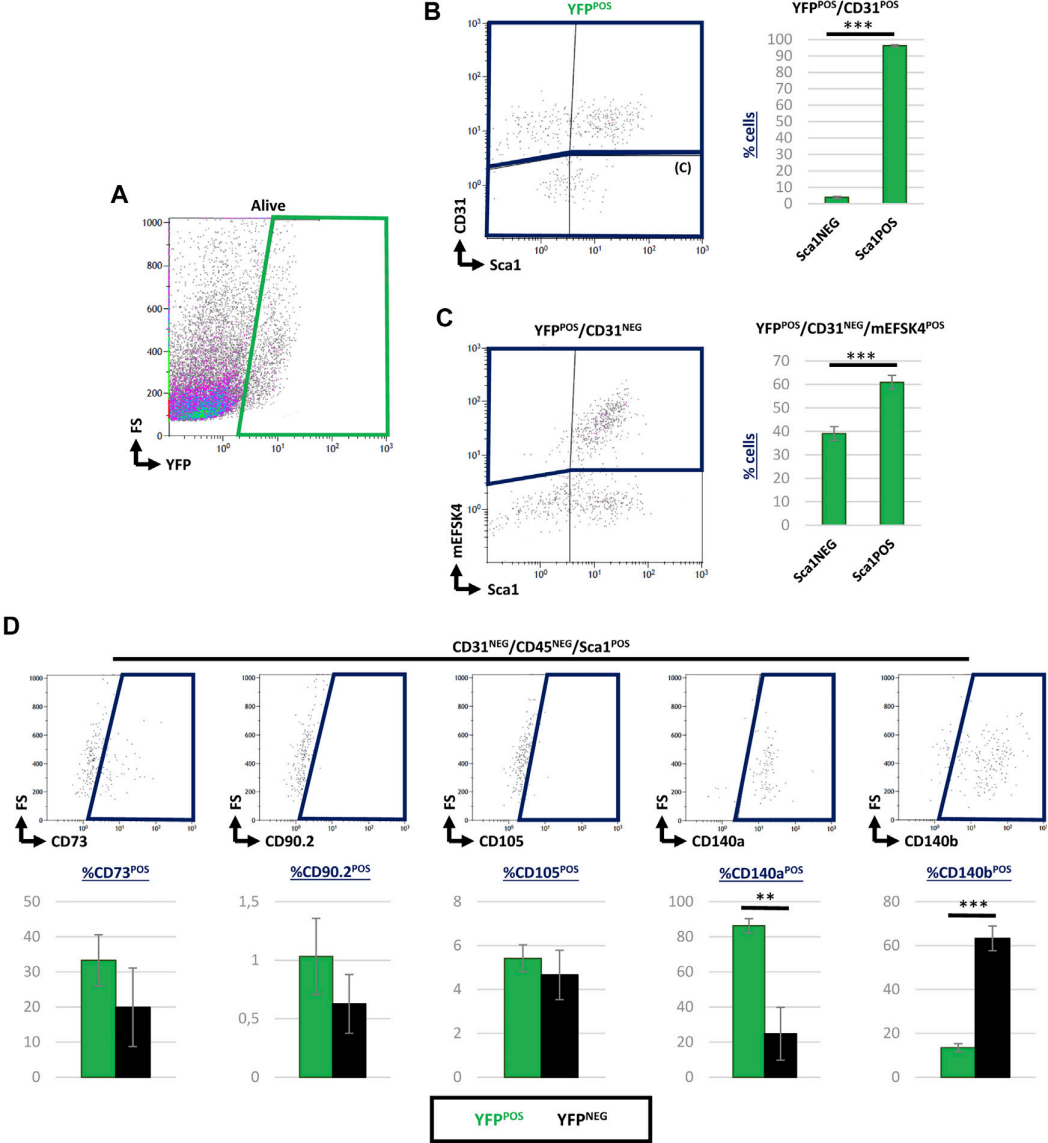
Contribution of Sca1^POS^ cells into different subpopulations of epicardial-derived cells in the adult heart. **(A)** Representative dot blots showing the analysis of YFP^POS^ cardiac cells. **(B)** Dot blot showing CD31 and Sca1 staining of YFP^POS^ cells (left). The bar plot represented the percentages of Sca1^NEG^ and Sca1^POS^ cells within the YFP^POS^/CD31^POS^ subpopulation (right). **(C)** Representative dot blots showing the staining of mEFSK4 and Sca1 within the YFP^POS^/CD31^NEG^ fraction (left). The bar plot representation of Sca1^NEG^ and Sca1^POS^ cells into percentages within the YFP^POS^/CD31^NEG^/mEFSK4^POS^ cardiac fibroblasts population (right). **(D)** Dot blots summarizing the results from CD73, CD90, CD105, CD140a, and CD140b staining of CD31^NEG^/CD45^NEG^/Sca1^POS^ cardiac interstitial cells stained. The bar plot indicates the percentage of these specific subpopulations within the YFP^POS^ (green bars) and YFP^NEG^ (black bars) cell fractions. Abbreviations: CD31/PECAM1, platelet and endothelial cell adhesion molecule 1; CD45, protein tyrosine phosphatase receptor type C; CD73, cluster of differentiation 73; CD90, Thy-1 cell surface antigen; CD105, endoglin; CD140a, platelet-derived growth factor receptor A; CD140b, platelet-derived growth factor receptor B; Sca1, stem cell antigen-1; YFP, yellow fluorescent protein. N, 3 biological replicates. **p-value ≤0.01; ***p-value ≤0.005.

Since a significant percentage of Sca1^POS^ CICs co-expressed YFP in the adult, we aimed at characterizing these cells in detail at this stage. We, therefore, searched for Sca1^POS^ cells within the epicardial lineage-derived endothelial and cardiac fibroblast populations (Acharya et al., 2012; Katz et al., 2012; Ruiz-Villalba et al., 2015; Cano et al., 2016). Considering the total YFP^POS^ cardiac cells (Figure 4A), our analysis revealed that more than 90% of Wt1 lineage-derived CD31^POS^ ventricular endothelial cells co-express Sca1 (Figure 4B; Table 3). Then, to determine the percentage of Sca1^POS^ cells in the cardiac fibroblasts (CFs) population we used the mEFSK4 antibody, a known CF pan-marker (Pinto et al., 2016; Ruiz-Villalba et al., 2020). Our analysis showed that around 60% of YFP^POS^/CD31^NEG^/mEFSK4^POS^ CFs also co-expressed Sca1 (Figure 4C; Table 3). These data clearly indicate that both the putative epicardial-derived endothelial and cardiac fibroblasts populations express Sca1.

**TABLE 3:** Percentage of Sca-1^POS^ cells included in both YFP^POS^ and YFP^NEG^ fractions into the different characterized subpopulations (mean ± s.e.m.).

YFP^POS^		
	CD31^POS^	CD31^NEG^
Sca1^POS^	47.59±3.42	27.22±0.56
Sca1^NEG^	1.9±0.4	23.29±4.30

YFP^POS^		
	CD31^NEG^/mEFSK4^POS^	CD31^NEG^/mEFSK4^NEG^
Sca1^POS^	46.15±7.35	8.30±4.83
Sca1^NEG^	30.08±8.25	15.56±11.64

CD31^NEG^/CD45^NEG^/Sca1^POS^		
	YFP^POS^	YFP^NEG^
CD73	33.32±7.29	19.91±11.16
CD90	1.03±0.32	0.63±0.25
CD105	5.42±0.61	4.66±1.12
CD140a	86.38±3.99	24.74±15.06
CD140b	13.45±1.77	63.32±5.65

To complete the characterization of Sca1^POS^/YFP^POS^ cells, we considered the potential of some Wt1 lineage-derived cells as mesenchymal progenitors, according to previously suggested (Wessels and Pérez-Pomares, 2004; Pogontke et al., 2019). In order to do so, we analyzed the CD31^NEG^/CD45^NEG^/Sca1^POS^ CICs population in terms of their expression of classical markers for mesenchymal progenitors such as CD73, CD90, CD105, CD140a, or CD140b (Dominici et al., 2006; Farahani and Xaymardan, 2015; Tang et al., 2020) (Figure 4D). This analysis revealed no significant differences between YFP^POS^ and YFP^NEG^ CICs regarding the expression of CD73, CD90, and CD105. However, 87% of CD31^NEG^/CD45^NEG^/Sca1^POS^/YFP^POS^ CICs co-expressed CD140a, and a 13% coexpressed CD140b (Table 3).

These results are in accordance with the recently described contribution of Sca1^POS^ cells into the cardiac interstitium (Tang et al., 2018, 2020). However, our results point out the heterogeneous nature of this subpopulation of adult Sca1^POS^ CICs and a close relationship with specific subpopulations of epicardial-derived lineages, such as cardiac endothelium and fibroblasts. This is again relevant in terms of understanding the cardiac interstitium in both homeostasis and response to pathologic stimuli.

### Postnatal Epicardial Cell Tracing Discards *De Novo* Contribution of This Cell Lineage Into the Adult Sca1^POS^ Interstitial Cell Subpopulation

**FIGURE 5:**
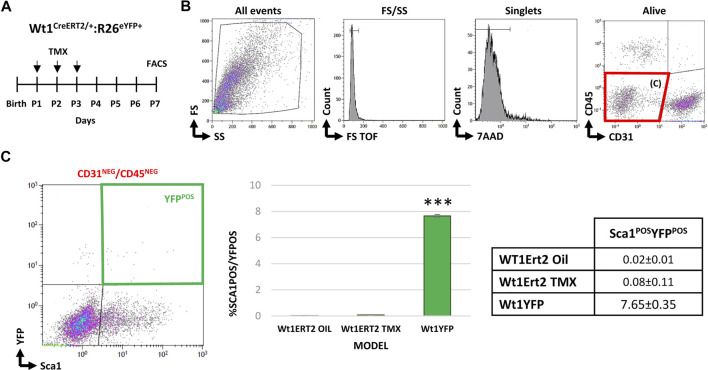
Studying *de novo* cardiac expression of *Wt1* after birth. **(A)** Wt1Cre-ERT2 mice were crossed with the Rosa26R-eYFP reporter line to conditionally induce the activation of the driver between P1 and P3. **(B)** Representative dot blots and cytograms showing the gating strategy followed to characterize CD31^NEG^/CD45^NEG^ cardiac interstitial subpopulations. **(C)** Bar plot representation of cardiac CD31^NEG^/CD45^NEG^/Sca1^POS^/YFP^POS^ cells in different experimental groups: Wt1Cre/ERT2-YFP injected with the vehicle (negative control); Wt1Cre/ERT2-YFP injected with tamoxifen as an experimental group; and Wt1Cre-YFP (positive control) (*n* = 12 peer group). The table shows the percentages of each subpopulation. Abbreviations: CD31/PECAM1, platelet, and endothelial cell adhesion molecule 1; CD45, protein tyrosine phosphatase receptor type; FS, forward scatter; Sca1, stem cell antigen-1; SS, side scatter; YFP, yellow fluorescent protein. ***p value ≤0.005.

In a final series of experiments, we aimed at discarding a potential *de novo* postnatal appearance of Sca1^POS^/YFP^POS^ CICs (between P1 and P3). To tackle this task, we crossed the conditional Wt1Cre^ERT2^ transgenic line with the Rosa26R-eYFP reporter one (Figure 5A). Our analysis revealed that YFP^POS^ cells were absent from the CD31^NEG^/CD45^NEG^ cardiac cells fraction at P7 (Figures 5B,C). These results supported an embryonic epicardial origin for the adult CD31^NEG^/CD45^NEG^/Sca1^POS^/YFP^POS^ CICs identified and characterized in this work.

## Discussion

Cardiac interstitial cells (CICs) are a dynamic and heterogeneous cell population. This poorly studied group of cells has been historically overshadowed by the size and functional relevance of cardiomyocytes. CICs, however, largely exceed cardiac muscle cells in number and their enormous importance in the diseased heart has recently attracted much attention. Indeed, CICs contain progenitors and quiescent forms of cardiac fibroblasts (CFs), which are central to the reparative fibrotic response that characterizes the pathophysiology of multiple cardiac diseases, from ischemic, dilated, and arrhythmogenic cardiomyopathies to the aging and diabetic heart. Increasing our understanding of this unique group of cells will open new avenues for the early diagnosis of and effective therapy for these ailments.

The ontogenetic origin of CICs has been shown to be very diverse, but two of the main sources for these cells have been identified, namely, the epicardial-derived mesenchymal cell progeny often referred to as epicardial-derived cells or EPDCs and the blood cell lineages (for a comprehensive review see Pogontke et al., 2019). In both cases, the cellular colonization of the interstitial space takes place progressively during the perinatal stages (Epelman et al., 2014; Moore-Morris et al., 2015; Ruiz-Villalba et al., 2015). The main aim of this study was to study two specific CICs fractions (c-Kit^POS^ and Sca1^POS^) in relation to epicardial-derived and hematopoietic/blood cell lineages. Since the beginning of this century, both c-Kit^POS^ and Sca1^POS^ have been considered to represent multipotent resident cardiac stem cell (CSC) populations (Beltrami et al., 2003; Vicinanza et al., 2017). This concept was first intensely debated and then recently refuted, so that the existence of this CSCs as they were originally described is no longer accepted (Van Berlo et al., 2014; Sultana et al., 2015). The existence of CICs expressing c-Kit and Sca1 remains, however, an undisputed fact, but their real nature is far from having been deciphered.

Our FACS analysis first revealed that very few c-Kit^POS^ cells are related to cardiac Wt1-lineage-derived cells, which can be genetically tagged by their original embryonic expression of the Wilms’ tumor suppressor gene 1 (Wt1). This analysis was specifically carried out in the CD31^NEG^/CD45^NEG^ CICs fraction to exclude endothelial and blood-borne cells from the screening. We nonetheless identified a small proportion of c-Kit^POS^/YFP^POS^ CICs whose identity is not still clear. The presence of c-Kit^POS^, EPDCs homologous mesothelial-derived Cajal cells in the intestinal tract (Carmona et al., 2013) strongly suggested that cardiac c-Kit^POS^/YFP^POS^ cells are not an artifact of the genetic cell tracing technology but a real biological entity. In any case, immunohistochemistry unambiguously shows that c-Kit^POS^/YFP^POS^ is normally located in the subepicardial space or in contact with the wall of coronary blood vessels, two domains massively contributed by epicardial cells (Cano et al., 2016). The sharp postnatal decrease in c-Kit^POS^/YFP^POS^ cell numbers within the CD31^NEG^/CD45^NEG^ CICs fraction observed by FACS may reflect a progressive dilution of a poorly proliferating cell subset with respect to other expanding cardiac cell types and also reflects the difficulties of finding significant numbers of these cells in adult cardiac tissues using immunohistochemical approaches. Alternatively, it could be argued that the decrease in the number of c-Kit^POS^/YFP^POS^ cells is due to the loss of this cell surface marker during cell differentiation/maturation. More research is needed to clarify this point.

As the majority of cardiac c-Kit^POS^ cells are also YFP^NEG^ in Wt1Cre-YFP mice, we then decided to evaluate a possible origin for these cells from hematopoietic/blood lineages. In order to do so, we searched for the specific pan-leukocyte marker CD45 in CICs. The results from this inspection show that CD45^POS^/c-Kit^POS^ cells are abundant among CICs (up to 65% at E15.5). As expected, virtually none of these cells was YFP^POS^, excluding a possible relation between blood-borne cells and Wt1 cell lineages. Of note, nearly all c-Kit^POS^ CICs co-expressed CD34, an immature endothelium and stem cell marker (Carmona et al., 2020). Furthermore, c-Kit^POS^ cells expressing the endothelial and hematopoietic cell marker CD31^POS^ were also identified in our immunohistochemical analysis. Other authors have already identified c-Kit^POS^/CD45^NEG^ CICs as endothelial progenitors in the adult (Sandstedt et al., 2010). However, as described above, we found a relevant contribution of c-Kit^POS^ cardiac cells in the CD45^POS^ CIC fraction, suggesting that these cells are therefore more likely to be blood-borne cells than endothelial cells. In accordance with this conclusion, CD45^NEG^/c-Kit^POS^/YFP^NEG^ CICs were found to encompass cells expressing markers for macrophages (F4/80), lymphoid (CXCR3), and myeloid (CD11b) cell types, a result that endorses an extracardiac origin for these CIC. Finally, a marked decrease in the number of CD45^POS^/c-Kit^POS^ CICs was observed between embryonic and postnatal stages, a finding that can be interpreted either as a cell dilution similar to that previously described for c-Kit^POS^/YFP^POS^ cells or as an actual decrease of blood circulating cell recruitment to the adult heart.

The second part of our study focused on the study of Sca1^POS^ CICs. FACS characterization of Wt1Cre-YFP CICs clearly indicates that the majority of CD31^NEG^/CD45^NEG^/Sca1^POS^ CICs were YFP^POS^ at P7, a finding that relates these cells with the Wt1-derived cardiac mesenchymal lineage. The progressive, sustained increase of Sca1^POS^ CICs from developmental to postnatal stages (rising from 0% to 2% to almost 70%) is evidence of the fast expansion of these cells after birth. Further FACS characterization indicates that an important number of these cells are CD31^POS^ endothelial ones (Tang et al., 2018, 2020). Indeed, we show that 30% of CD31^POS^ ventricular endothelial cells also expressed Sca1 at E18.5, reaching 80% in the adult, indicating that the contribution of Sca1^POS^/CD31^POS^ cells to the cardiac interstitium is highly dynamic over time. Relevant to this discussion, 90% of YFP^POS^/CD31^POS^ cells and 60% of YFP^POS^/CD31^NEG^/mEFSK4^POS^ CFs expressed Sca1, suggesting that the expression of this molecule is related to the epicardial cell lineages. This finding could be relevant due to the importance of epicardial cells in the context of cardiac repair (Ruiz-Villalba et al., 2015; Chen et al., 2018). However, since Sca1^POS^/YFP^POS^ and Sca1^POS^/YFP^NEG^ cells included similar percentages of mesenchymal stem-like cells, such as CD73, CD90, and CD105, more research is needed to determine potential differences between both cell subpopulations.

We have shown that postnatal CD31^NEG^/CD45^NEG^/Sca1^POS^/YFP^POS^ CICs are likely to arise through the proliferation of prenatal cell populations, although the possibility of this increase results from the *de novo* expression of Wt1 in the Sca1^POS^ population cannot be directly discarded. To ensure this was not the case, we used an inducible epicardial driver line (Wt1Cre^ERT2^) to activate the promoter in the postnatal stages. At P7, we did not identify YFP^POS^ CICs, confirming that CD31^NEG^/CD45^NEG^/Sca1^POS^/YFP^POS^ CICs derive from embryonic founder cells.

Our study provided novel evidence on the relationship existing between cardiac interstitial c-Kit^POS^ and Sca1^POS^ cell populations and the Wt1 cell lineage, also contributing relevant information on the spatiotemporal distribution of these cells in the embryonic and postnatal heart. Since the majority of cardiac Wt1 lineage cells (E11-E17) are known to derive from the embryonic epicardium (Wessels et al., 2012; Cano et al., 2016), we generally assumed that YFP cells are epicardial derivatives. In accordance with this view, recent publications based on single-cell RNA-Seq revealed the existence of an epicardial transcriptomic signature related to Wt1 lineage-derived cells in the adult heart (Quijada et al., 2020; Hesse et al., 2021). For this reason, we considered that adult YFP cardiac cells are mostly derived from the embryonic epicardium. Notwithstanding, this conclusion does not rule out the possibility of Wt1 lineage cells deriving from other alternative cellular sources (King-Underwood et al., 2005).

As it can be inferred from the previous discussion, Sca1^POS^/YFP^POS^ CICs are very likely to be epicardial derivatives. The enrichment of Sca1 in a major part of adult putative epicardial-derived cells suggested that this molecule may be involved in the regulation of epicardial biology. The colonization of the cardiac interstitium by these cells starts during embryonic development and is highly dynamic. In this respect, since the turnover rate for CICs is unlikely to be steady throughout adult life, the specific cellular dynamics and composition of the cardiac interstitium have been proposed to be a sensor for the health status of the heart (Pogontke et al., 2019).

In summary, increasing our knowledge of the origin, diversity, and functions of CICs will be instrumental to the development of a diagnostic and prognostic test for the evaluation of cardiac homeostasis and the cardiac interstitium´s response to pathologic stimuli. However, further, systematic research on CICs biology is needed to bring about significant progress in our understanding of interstitial responses to pathologic conditions.

## Data Availability

The raw data supporting the conclusion of this article will be made available by the authors, without undue reservation.

## Supplementary Material

Supplementary Figure S1 Negative controls for FACS experiments. Representative histograms showing the samples without primary antibody (dark blue) *vs*. samples with specific antibodies of interest (light blue) analyzed at developmental, postnatal, and adult stages **(A)**, and others that have been only analyzed in adulthood **(B)**. Abbreviations: CCR2, C-C motif chemokine receptor 2; CD11b, cluster of differentiation 11; CD31/PECAM1, platelet and endothelial cell adhesion molecule 1; CD34, cluster of differentiation 34; CD45, protein tyrosine phosphatase receptor type C; CD73, cluster of differentiation 73; CD90, Thy-1 cell surface antigen; CD105, endoglin; CD140a, platelet-derived growth factor receptor A; CD140b, platelet-derived growth factor receptor B; c-Kit, KIT proto-oncogene receptor tyrosine kinase; CXCR3, C-X-C motif chemokine receptor 3; F4/80, EGF-like module-containing mucin-like hormone receptor-like 1; Sca1, stem cell antigen-1.

Supplementary Figure S2 Characterization of c-Kit expression in cardiac blood-borne and endothelial cells. **(A)** Representative dot blot showing the gating for CD45^POS^ cells isolated from the heart. The cells were characterized based on their YFP and c-Kit expression (left). The bar plots represent the percentages of YFP^POS^ (green bars, middle) and YFP^NEG^ (black bars, right) cells within the CD45^POS^/c-Kit^POS^ subpopulation (E13.5, E15.5, E18.5, P1, P7, and adult) (right). **(B)** Representative dot blots showing the presence of YFP and c-Kit in the CD31^POS^ cardiac endothelial cell fraction(left). Bar plots summarize the percentages of YFP^POS^ (green bars) and YFP^NEG^ (black bars) cells within the CD31^POS^/c-Kit^POS^ cell subpopulation at the different time points indicated. **(C)** Immunolocalization of c-Kit and CD45 in Wt1Cre-YFP murine hearts between E13.5 and adulthood. CD45^POS^/c-Kit^NEG^ (white), CD45^NEG^/c-Kit^POS^ (red, arrowheads), and CD45^POS^/c-Kit^POS^ (light red, arrows) cells were located in the subepicardial region of the heart in embryonic and early postnatal stages, close to large coronary vessels (adult). No CD45^POS^/c-Kit^POS^/YFP^POS^ cells were identified. **(D)** Immunolocalization of c-Kit and CD31 in Wt1Cre-YFP murine hearts between E13.5 and E18.5. CD31^POS^/c-Kit^NEG^ (white), CD31^NEG^/c-Kit^POS^ (red, arrowheads), and CD31^POS^/c-Kit^POS^ (light red, arrow) cells were located in the subepicardial region of the embryonic hearts. No CD31^POS^/c-Kit^POS^/YFP^POS^ were found in any of the analyzed samples (*n* = 2 embryos from two different litters; or 2 individuals). Abbreviations: CD31/PECAM1, platelet, and endothelial cell adhesion molecule 1; CD45, protein tyrosine phosphatase receptor type; c-Kit, KIT proto-oncogene receptor tyrosine kinase; V, the lumen of coronary vessels; YFP, yellow fluorescent protein. Scale bars: 25 μm * = *p*-value ≤0.05; ***p*-value ≤0.01; ****p*-value ≤0.005.

Supplementary Figure S3 Characterization of Sca1^POS^ cells expression in cardiac blood-borne and endothelial cells. **(A)** Representative dot blots showing the gating applied to CD45^POS^ cardiac cells. The cells were characterized based on their YFP and Sca1 expression (left). The bar plots show the percentages of YFP^POS^ (green bars) and YFP^NEG^ (black bars) cells within the CD45^POS^/Sca1^POS^ cell subpopulation (E13.5, E15.5, E18.5, P1, P7, and adult) (right). **(B)** Representative dot blots showing the presence of YFP and Sca1 in the CD31^POS^ cardiac endothelial cell compartment (left). The bar plots represented the percentages of YFP^POS^ (green bars) and YFP^NEG^ (black bars) within the CD31^POS^/Sca1^POS^ cell fraction(right). **(C)** Immunolocalization of SCA1 cells in the adult Wt1Cre-YFP heart. Both Sca1^POS^/YFP^NEG^ (red) and Sca1^POS^/YFP^POS^ (yellow) located in the perivascular region of big coronary vessels. Some of these cells co-localized with lectin IB4 but not with αSMA (arrow). Abbreviations: αSMA, alpha-smooth muscle actin; CD31/PECAM1, platelet and endothelial cell adhesion molecule 1; CD45, protein tyrosine phosphatase receptor type; lectin, lectin isolated from *Griffonia simplicifolia* (IB4); Sca1, stem cell antigen-1; V, the lumen of coronary vessels; YFP, yellow fluorescent protein. Scale bars: 25 μm ***p*-value ≤0.01; ****p*-value ≤0.005.
