# Supplementary material for: Dynamic Epicardial Contribution to Cardiac Interstitial c-Kit and Sca1 Cellular Fractions
Source: Front Cell Dev Biol. 2022 May 30;10:864765. doi: 10.3389/fcell.2022.864765 (PMC9189417; doi:10.3389/fcell.2022.864765)
Supplement: Supplementary file 1 [file Table1.docx]

**Table M1**. Primary antibodies used in flow cytometry.

| Epitope | Host | Dilution | Reference | |
| --- | --- | --- | --- | --- |
| CD11bAf700 | Rat (M1/70) | 1:250 | Ebioscience | 56-0112-80 |
| CD31APC | Rat (MEC13.3) | 1:250 | Ebioscience | 17-0311-82 |
| CD34Af700 | Rat (RAM34) | 1:150 | BDBioscience | 560518 |
| CD45PE | Rat (30F11) | 1:250 | EBioscience | 12-0451-82 |
| CD146PE | Rat (SA023G11) | 1:150 | Biolegend | 150605 |
| CCR2BV421 | Rat (ME-9F1) | 1:200 | Biolegend | 134703 |
| c-KitAPC-780 | Rat (2B8) | 1:200 | Ebioscience | 47-1171-80 |
| CXCR3PE-Cy7 | Hamster (173) | 1:500 | Biolegend | 126515 |
| F4/80Af660 | Rat (BM8) | 1:200 | Ebioscience | 50-4801-80 |
| Sca1SB436 | Rat (D7) | 1:150 | Ebioscience | 62-5981-80 |
| CD73 | Rat (eBioT4/118) | 1:150 | Ebioscience | 12-0731-81 |
| Feeder Cells Ab | Rat (mEF-SK4) | 1:150 | Miltenyi | 130-120-802 |
| CD90.2 | Rat (30H12) | 1:150 | Ebioscience | 12-0903-82 |
| CD105 | Rat (MJ7/18) | 1:150 | Ebioscience | 12-1051-81 |
| PDGFRA | Rat (APA5) | 1:150 | Ebioscience | 17-1401-81 |
| PDGFRB | Rat (APB5) | 1:150 | Ebioscience | 12-1402-80 |
