# Supplementary figures and images for: Dynamic Epicardial Contribution to Cardiac Interstitial c-Kit and Sca1 Cellular Fractions
Source: Front Cell Dev Biol. 2022 May 30;10:864765. doi: 10.3389/fcell.2022.864765 (PMC9189417; doi:10.3389/fcell.2022.864765)

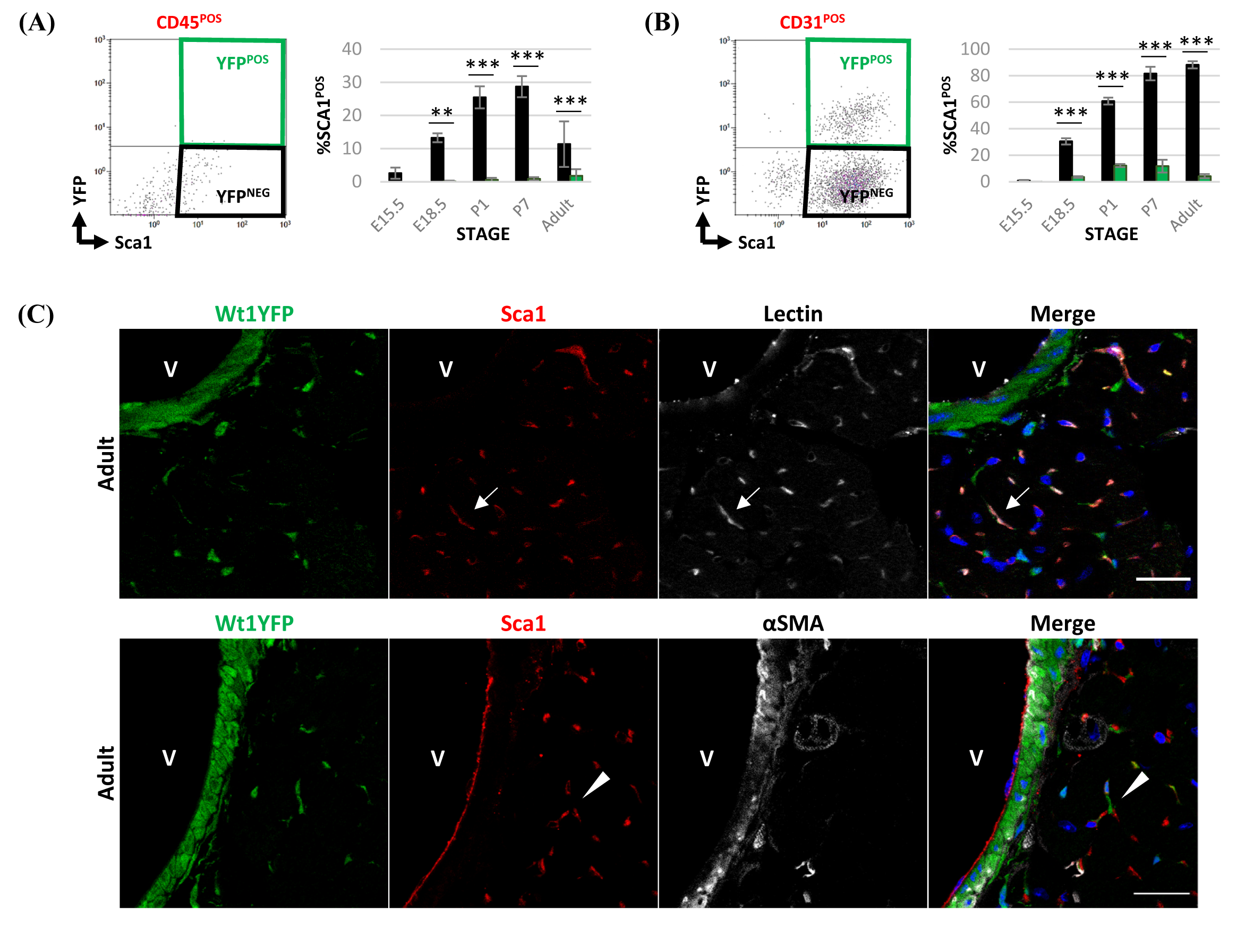

Supplement: Supplementary file 2 [file Image3.TIF]

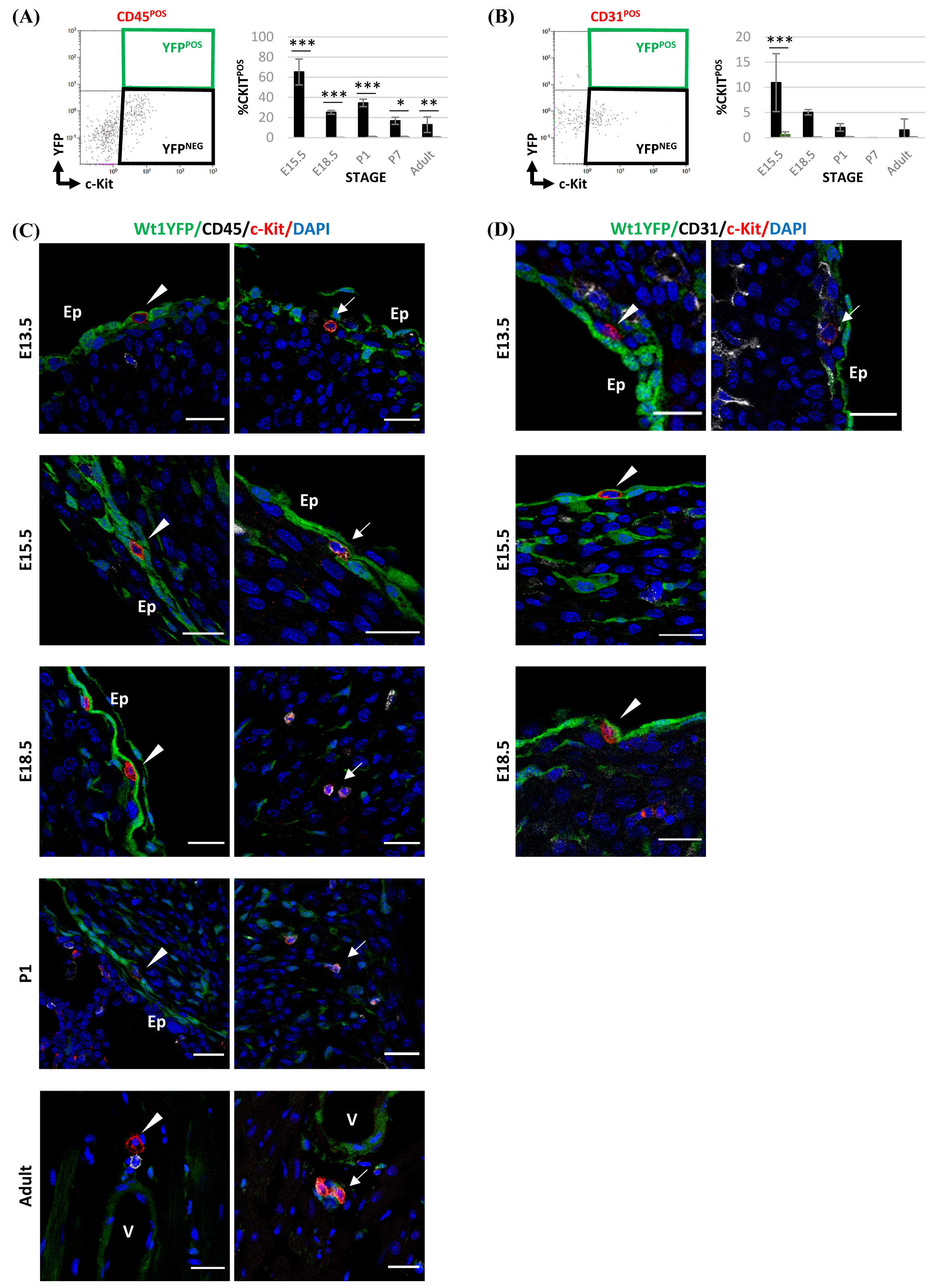

Supplement: Supplementary file 3 [file Image2.TIF]

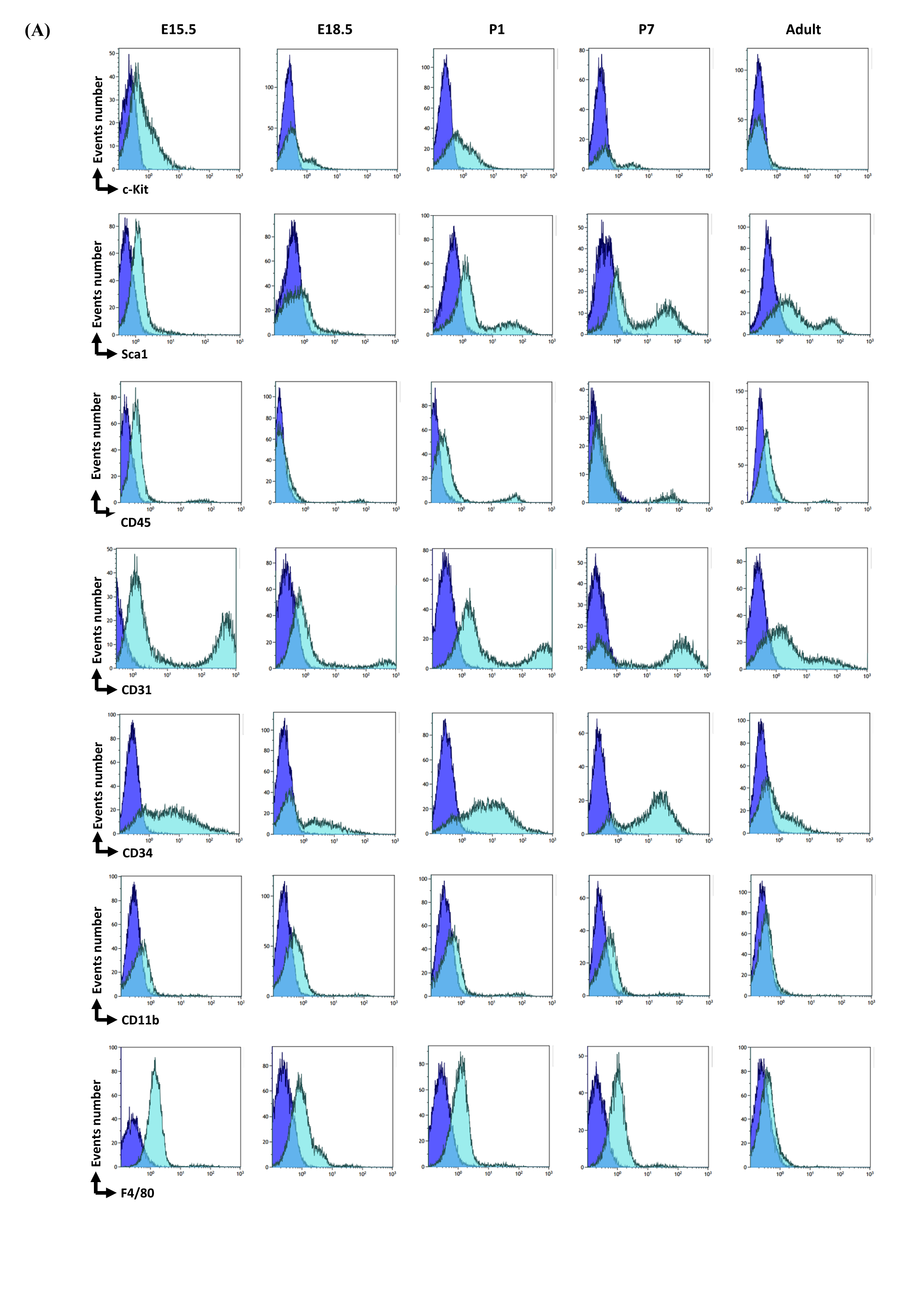

Supplement: Supplementary file 4 [file Image1.TIF]
