# Supplementary material for: Dynamic Epicardial Contribution to Cardiac Interstitial c-Kit and Sca1 Cellular Fractions
Source: Front Cell Dev Biol. 2022 May 30;10:864765. doi: 10.3389/fcell.2022.864765 (PMC9189417; doi:10.3389/fcell.2022.864765)
Supplement: Supplementary file 5 [file Table2.docx]

**Table M2**. Primary antibodies used in immunohistochemistry.

| Epitope | Host | Dilution | Reference | |
| --- | --- | --- | --- | --- |
| αSMA | Rabbit | 1:100 | Abcam | Ab5694 |
| αSMA | Mouse | 1:100 | Sigma | A2518 |
| βIIITubulin | Mouse | 1:100 | Biolegend | 801213 |
| CD31 | Rat (MEC13.3) | 1:100 | BDPharmingen | 550274 |
| CD45APC | Rat (30F11) | 1:100 | Miltenyi | 130-097-962 |
| c-Kit | Goat | 1:100 | RyDSystem | Af1356 |
| LectinTRITC | -------- | 1:100 | Sigma | L5294 |
| Sca1Af647 | Rat (E-13) | 1:100 | Biolegend | 122518 |
| Sca1Af647 | RatD7 | 1:100 | Biolegend | 108117 |
