# Supplementary material for: Dynamic Epicardial Contribution to Cardiac Interstitial c-Kit and Sca1 Cellular Fractions
Source: Front Cell Dev Biol. 2022 May 30;10:864765. doi: 10.3389/fcell.2022.864765 (PMC9189417; doi:10.3389/fcell.2022.864765)
Supplement: Supplementary file 7 [file Table3.docx]

**Table M3**. Secondary antibodies used in immunohistochemistry.

| Epitope | Host | Dilution | Reference | |
| --- | --- | --- | --- | --- |
| GoatFITC | Rabbit | 1:400 | Sigma | B7367 |
| GoatTRITC | Donkey | 1:400 | Jackson | 705-025-147 |
| GoatCy5 | Donkey | 1:400 | Jackson | 705-175-147 |
| MouseFITC | Goat | 1:400 | Sigma | F2012 |
| MouseTRITC | Goat | 1:400 | Sigma | T5393 |
| MouseAf647 | Donkey | 1:400 | Jackson | 715-605-151 |
| RabbitFITC | Goat | 1:400 | Sigma | F9887 |
| RabbitTRITC | Goat | 1:400 | Sigma | T6778 |
| RabbitCy5 | Donkey | 1:400 | Jackson | 711-605-152 |
| RatAf488 | Donkey | 1:400 | Jackson | 712-545-153 |
| RatTRITC | Donkey | 1:400 | Jackson | 712-025-153 |
| RatCy5 | Donkey | 1:400 | Jackson | 712-605-150 |
